# Identification of different carbenium ion intermediates in zeolites with identical chabazite topology *via*^13^C–^13^C through-bond NMR correlations†

**DOI:** 10.1039/c9ra02280e

**Published:** 2019-04-23

**Authors:** Dong Xiao, Xiuwen Han, Xinhe Bao, Guangjin Hou, Frédéric Blanc

**Affiliations:** State Key Laboratory of Catalysis, Dalian Institute of Chemical Physics, Chinese Academy of Sciences 457 Zhongshan Road Dalian 116023 China; University of Chinese Academy of Sciences Beijing 100049 China; Department of Chemistry, University of Liverpool Crown Street Liverpool L69 7ZD UK frederic.blanc@liverpool.ac.uk; Stephenson Institute for Renewable Energy, University of Liverpool Crown Street Liverpool L69 7ZD UK

## Abstract

^13^C–^13^C through-bond NMR correlation experiments reveal the stabilization of different carbenium ion intermediates in two zeolites possessing identical CHA topology (H-SAPO-34 and H-SSZ-13) during the methanol to olefins reaction.

## Abstract 2

Multidimensional NMR experiments explicitly distinguish differences in carbenium ion intermediates stabilized in two zeolites with identical topology.

## Graphical abstract

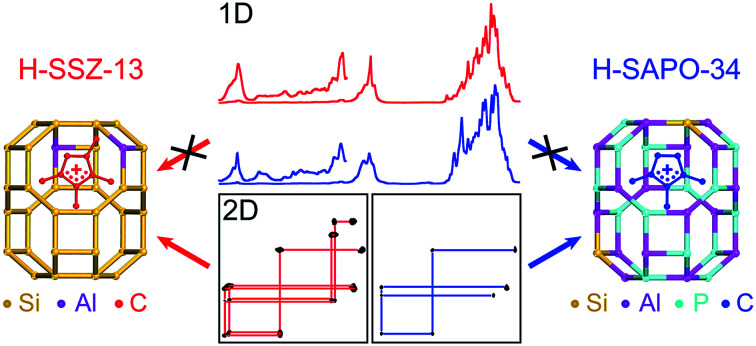

The production of light olefins *via* the methanol-to-olefins (MTO) reaction is an important chemical process that links non-oil resources such as coal and natural gas with olefin-based petrochemicals.^1–5^ The catalysts used for the MTO reaction are mainly microporous acidic zeolites amongst which H-SAPO-34, a silicoaluminophosphate zeolite with the chabazite (CHA) topology, is of particular importance due to its high selectivity to ethylene and propene, and is of commercial use.^1,3^ H-SSZ-13 is a CHA silicoaluminate analogue of H-SAPO-34 which has been shown to be a potential alternative in the MTO process.^6^

Despite the successful industrialization of this process with methanol conversion higher than 99%,^3^ further improving of the catalyst performances in terms of selectivity has been an important scientific endeavor. For example, the selectivity to ethylene and propene has increased from 79.2% in the first generation industrial DMTO process (“D” refers to Dalian Institute of Chemical Physics) to 85.7% in the second generation DMTO-II process in China.^3^ However, there is still significant room to improve catalysts performances which can be informed by providing a deeper understanding of the catalytic reaction intermediates and reaction mechanism.

We and others have previously investigated the MTO mechanism on H-ZSM-5,^7–10^ H-SAPO-34 ^11–14^ β zeolite^15,16^ using a range of experimental and computational approaches including solid-state Nuclear Magnetic Resonance (NMR). The hydrocarbon pool (HCP) mechanism has been generally accepted for the formation of hydrocarbons from methanol^5,11,12,17,18^ and suggests that for the aromatic cycle routes the organic species (mainly cyclic carbenium ions and neutral aromatic species) confined in the pores of zeolites act as co-catalysts with the inorganic framework. Based on the species observed, a side-chain and a paring reaction pathways have been proposed. While the former involves olefins released through methylation of six-membered ring cations (alkylbenzenium) and elimination of the side chain groups, the later route produces the olefins *via* expansion of alkylcyclopentenyl cations followed by contraction of the formed six-membered ring cations.^14,15,19^ These cyclic carbenium ions are key nodes along the reaction routes and their identification plays an important role in determining the mechanism acting for a given zeolite.

Solid-state NMR has played a critical role in achieving this understanding^8,14,20,21^ and our recent works^10,16^ deploying a range of multidimensional and multinuclear NMR approaches have enabled the unequivocal structural identification of a range of five- and six-membered ring cations (as well as neutral compounds) in H-ZSM-5 and β-zeolites (MFI and BEA topologies, respectively), without the need for previous knowledge or assumption of the carbenium ions structures. In particular, this approach permits to distinguish carbenium ions with closely related structures (*e.g.*, cyclopentenyl cations with various methyl groups) and previously unidentified carbenium ions (*e.g.*, 1,5-dimethyl-3-*sec*-butyl cyclopentenyl cation and methylnaphthalenium cations), all offering a complete understanding of the reaction routes.

Here, we probe the carbenium ions formed during the MTO reaction on two different CHA zeolites (H-SSZ-13 and H-SAPO-34) by utilizing the 2D ^13^C–^13^C refocused INADEQUATE (Incredible Natural Abundance DoublE QUAntum Transfer Experiment)^22^ NMR experiment (pulse sequence in Fig. S1 in the ESI†). We experimentally identified 1,2,3,4-tetramethylcyclopentenyl(I) and 1,2,3-trimethylcyclopentenyl(II) cations as the major retained cation species on H-SSZ-13 and H-SAPO-34 CHA zeolites, respectively.

Activated zeolites were prepared by flowing ^13^C enriched CH_3_OH on H-SSZ-13 and H-SAPO-34 catalyst beds at 275 °C for 25 min and 300 °C for 20 min, respectively, followed by quenching into liquid N_2_ to capture the carbenium ion intermediates (full experimental details are given in the ESI†). The ^13^C CP (Cross Polarisation) MAS (Magic Angle Spinning) NMR spectra of these two activated catalysts are given in Fig. 1 and show multiple signals in the 0–260 ppm region, highlighting the complexity of the retained carbon species. Three general type of species (carbenium ions, aromatics/dienes, adamantane derivatives, see ESI†) are present, the downfield signals above 150 ppm being characteristic of carbenium ions.^8,10,14,16,21^ It is worth pointing out that the ^13^C spectra of activated H-SSZ-13 and H-SAPO-34 are very similar in this high frequency region; the two main peaks at about 153 and 243 ppm are representative signals for polymethylcyclopentenyl cations.^8,14,20,21^ These 1D NMR spectra would suggest that the same cations are retained on these two zeolites with identical CHA topology, however the more informative 2D through-bond ^13^C–^13^C correlation NMR experiment^22^ reveals that this is not the case (see below).

**Fig. 1:**
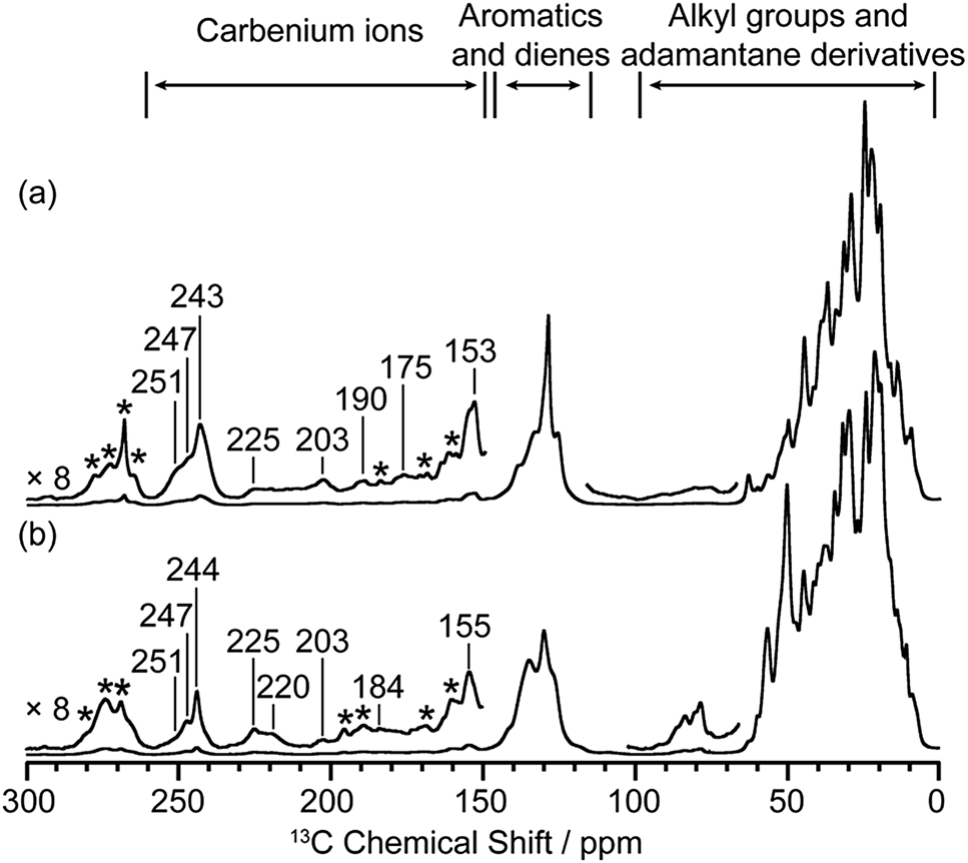
^13^C CP MAS spectra of activated (a) H-SSZ-13 and (b) H-SAPO-34. Spectra were recorded at 9.4 T and at a MAS of 14 kHz. Only characteristic signals for carbenium ions are labelled with chemical shifts. Asterisks (*) denote spinning sidebands (see Fig. S2 and S3†).

The corresponding 2D ^13^C–^13^C refocused INADEQUATE spectra of both activated zeolites are shown in Fig. 2. These experiments are based on through-bond scalar J coupling^22^ (rather than through-space dipolar-based experiments)^23,24^ and the correlation maps directly yield C–C bonds information, unambiguously enabling assignment of the carbon resonances. In this experiment, two peaks resonating at the same frequency in the double quantum (vertical) dimension arise from the sum frequency of the two individual ^13^C peaks in the single quantum (horizontal) dimension that correspond to chemically bonded carbons. The 2D spectra of the two activated zeolites show that the ^13^C–^13^C correlation maps are distinct as evidenced by correlations of the 63 and 243 ppm signals in H-SSZ-13 and of the 47 and 244 ppm peaks in H-SAPO-34 (see red and blue traces in Fig. 2), demonstrating that the main carbenium ions formed are different in both zeolites with the same CHA topology. The full maps are also shown in the 2D spectra and reveal experimental observation of the characteristic correlations C1(I) (243 ppm)–C2(I) (153 ppm) and C2(I) (153 ppm)–C3(I) (251 ppm) identifying cation I as the main retained cation species in H-SSZ-13 (Fig. 2a) and C1,3(II) (244 ppm)–C2(II) (155 ppm) and C1,3(II) (244 ppm)–C4,5(II) (47 ppm) for cation II in H-SAPO-34 (Fig. 2b and S4–S6† for complete set of correlations for each cation), allowing the structure of the carbenium ions and their ^13^C chemical shifts to be explicitly obtained (Table 1).

**Fig. 2:**
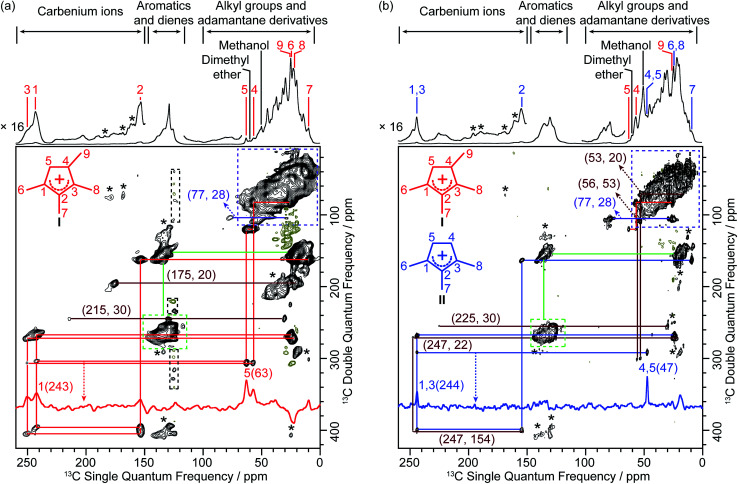
2D ^13^C–^13^C refocused INADEQUATE spectra of activated (a) H-SSZ-13 and (b) H-SAPO-34. Data were recorded at 9.4 T and at a MAS rate of 14 kHz. Signals of positive intensities and Fourier Transform (FT) wiggles of negative intensities are coded in black and olive, respectively. Asterisks (*) denote spinning sidebands. The assignments of the different carbenium ions and their corresponding structures are coloured-coded. Some representative traces extracted along the horizontal dimension are also shown. The complete set of traces for carbenium ions I and II is given in Fig. S4–S6.† Partial correlations for other carbenium ions are coded in maroon and displayed in Fig. S7.† The correlations coded in green and purple belong to the neutral species (aromatics, dienes and adamantane derivatives) (Fig. S8†). Signals off the carrier frequency in black dashed box correspond to small artefacts caused by direct current (DC) offset. Numbers in parenthesis are the chemical shifts of the correlated ^13^C sites.

**Table 1:** ^13^C chemical shifts for carbenium ions I and II (structures are shown in Fig. 2)

Carbon number		C1	C2	C3	C4	C5	C6	C7	C8	C9
Chemical shift/ppm	I	243	153	251	57	63	25	10	22	26
	II	244	155	244	47	47	24	9	24	—a

a Not applicable.

Note that the tetramethyl substituted cyclopentenyl cation I has not been observed in the literature.^8,10,14,21,25,26^ While trimethyl substituted cyclopentenyl II has been previously postulated in a previous study on H-SAPO-34 (ref. 27) based solely on 1D ^13^C spectra, it is worth pointing out that the 2D NMR method adopted here yields C–C bonding information directly, providing straightforward structural identification not accessible using methods that require treatment of post-activated zeolites.^8,14,26,28^

Additional correlations involving ^13^C signals in the high frequency range (175 to 250 ppm) are also observed (maroon lines in Fig. 2 and S7†). The correlations between signals at 30–215 ppm in H-SSZ-13 and 30–225 ppm in H-SAPO-34 are assigned to polymethylcyclohexenyl cations.^10,27,29^ In H-SAPO-34, the 247 ppm resonance shows multiple correlations involving carbenium ions which are most likely other cyclopentenyl cations.^8,14,26^ However, only a limited number of correlations is obtained, due to their low concentration as evidenced by their weak 1D signal intensities (Fig. 1), and challenges a complete structural determination for these cations. In H-SSZ-13, there is a unique correlation between signals at 20 and 175 ppm and is characteristic of methylnaphthalenium cations,^16^ the formation of this coke precursor being ascribed to the higher acid strength of H-SSZ-13 than H-SAPO-34 (as determined by infrared spectroscopy studies^6,30^ and a combination of ^1^H NMR, acetone probed ^13^C NMR and NH_3_-temperature programmed desorption methods).^14^ The signals in the 190–203 ppm region in the 1D spectra of both zeolites (Fig. 1, S2 and S3†) arise from polymethylbenzenium cations^14^ but are absent in the 2D spectra likely due to their very low concentration too.

Whilst CP MAS-based experiments like the refocused INADEQUATE are inherently not quantitative, the similar natures of the carbenium ions stabilized permit some consideration regarding the main species present. It is found that I dominates the 2D spectrum in H-SSZ-13 while it is a minor retained cation species in H-SAPO-34 where II is the main cation. Note that only a few characteristic correlations corresponding to I has been detected in the later zeolite (*e.g.*, C4(I)–C5(I) and C4(I)–C9(I), as shown in Fig. 2b and S6†) as the others are likely beyond the detection limit of the 2D experiment. The difference in population of the main carbenium ions I and II present in these two zeolites probably arises from their different acid strengths. It is known that whether or not a carbenium ion could be persistently stabilized inside zeolites depends on its acid strength relative to that of the zeolites.^14,31–33^ The distribution of carbenium ions in H-SSZ-13 *vs.* H-SAPO-34 may suggest that I is less stable than II and requires stronger acid sites to be stabilized.

The observation of the five-membered ring cations I and II is also strong indication of the existence of paring route in zeolites of CHA topology. It is clear that the five-membered ring cations are the main retained carbenium ion intermediates in both H-SSZ-13 and H-SAPO-34, while the six-membered ring cations are only present as minor species. This result is consistent with previous observation^14^ showing that six-membered ring cations have higher activity and further transform and are therefore less likely to be observed than the five-membered ring cations in the CHA zeolites.

In addition to carbenium species, neutral aromatics and dienes are present (correlated signals in the 115–150 ppm and 10–25 ppm regions, green dashed boxes and lines in Fig. 2 and S8†) and are involved as HCP species in the catalytic cycles and precursors of carbenium ions, respectively.^27^ Correlations in the 10–50 ppm region (purple dashed boxes in Fig. 2 and S8†) are assigned to alkyl groups (in aromatics and carbenium ions) and methyladamantanes, the later being consistent with gas chromatography – mass spectrometry experiments performed on CHA zeolites and suggested as coke species leading to catalyst deactivation.^34^ A correlation between signals at 77 and 28 ppm (purple lines in Fig. 2 and S8†) is also obtained on both zeolites and likely arises from hydroxy and methoxy substituted adamantanes.^35^ Note that the poor resolution of the 2D spectra in these regions hinders complete assignments of the neutral species mentioned above. Finally, signals at 50 and 60 ppm do not display 2D correlations which is consistent with their assignments to strongly adsorbed methanol and dimethyl ether, respectively.^8,10^

In conclusion, we have explicitly obtained the molecular structures of the reactive carbenium ions in two zeolites with identical CHA topology using multidimensional through-bond NMR experiments. New types of polymethylcyclopentenyl cations are identified and may serve as crucial intermediates in the paring route for MTO reaction. The new cations identified here offer a more comprehensive understanding of the reaction routes and will inspire future researches on their roles in MTO processes.

## Conflicts of interest

There are no conflicts of interest to declare.

## Supplementary Material

RA-009-C9RA02280E-s001
