# Supplementary material for: Identification of different carbenium ion intermediates in zeolites with identical chabazite topology via13C–13C through-bond NMR correlations
Source: RSC Adv. 2019 Apr 23;9(22):12415–8. doi: 10.1039/c9ra02280e (PMC9063671; doi:10.1039/c9ra02280e)
Supplement: RA-009-C9RA02280E-s001 [file RA-009-C9RA02280E-s001.pdf]

Electronic Supplementary Material (ESI) for RSC Advances.
This journal is © The Royal Society of Chemistry 2019

Supplementary Information for:

Identification of different carbenium ion intermediates in zeolites with identical chabazite topology via ^{13}C-^{13}C through-bond NMR correlations

Dong Xiao,[abc] Xiuwen Han,[a] Xinhe Bao,[a] Guangjin Hou,[a] and Frédéric Blanc*[cd]

[a] State Key Laboratory of Catalysis, Dalian Institute of Chemical Physics, Chinese Academy of Sciences, 457 Zhongshan Road, Dalian 116023 (China)

[b] University of Chinese Academy of Sciences, Beijing 100049 (China)

[c] Department of Chemistry, University of Liverpool, Crown Street, Liverpool, L69 7ZD (UK)

[d] Stephenson Institute for Renewable Energy, University of Liverpool, Crown Street, Liverpool, L69 7ZD (UK)

* To whom correspondence should be addressed (F.B.).

E-mail: frederic.blanc@liverpool.ac.uk.

Experimental Details

Procedures for preparing the MTO (Methanol To Olefins reaction) activated zeolites

Templated SAPO-34 (Al + P)/Si ≈ 7) with triethylamine was purchased from Nankai University Catalyst Co., Ltd and H-SSZ-13 (Si/Al = 15) was provided by BASF.[1] The templated SAPO-34 zeolite was calcined in air at 550 °C for 6 h to remove the organic template and generate the H-type zeolite. Both H-SAPO-34 and H-SSZ-13 zeolites with particles in the 40 ~ 60 mesh range were placed in a fixed-bed quartz tubular reactor and dehydrated first at 500 ^{o}C under a continuous helium (99.999%, Dalian Special Gases Co., Ltd.) flow for 2 h. The temperature was then gradually decreased to the reaction temperature as follow. H-SAPO-34 and H-SSZ-13 were then reacted with $^{13}CH_3OH$ (99 atom % ^{13}C, Sigma-Aldrich) with a weight hourly space velocity (WHSV) of 2 h^{-1} at 300 °C for 20 minutes and at 275 °C for 25 minutes, respectively. Liquid N_2 was then used to quench the reaction by immersing the reactor into it, the input gas switched to helium and once cooled to room temperature, the reactor was transferred to a glove box protected by N_2 for storage. The activated zeolites were packed into NMR rotors quickly in air at room temperature.

NMR experimental details

The NMR experimental details for activated H-SSZ-13 and H-SAPO-34 are shown in Table S1 and S2, respectively. The data were processed using the TopSpin3.2 NMR software.

Table S1. NMR spectra acquisition parameters for activated H-SSZ-13

Experiment		^{13}C CP[a]		^{13}C CP refocused INADEQUATE[a]
Magic angle spinning rate / kHz	8	12.5	14	14
Number of scans	2048	2048	4096	192
Recycle delay / s	3	3	3	3
^{1}H rf[b] field for 90° pulse / kHz	70	70	70	70
CP contact time / ms	2	2	2	2
^{1}H rf amplitude ramp for contact pulse / kHz	ramp70100.100[c]	ramp70100.100	ramp70100.100	ramp70100.100
^{1}H rf field during contact pulse / kHz	60	60	60	60
^{13}C rf field during contact pulse / kHz	60	60	64	46
^{1}H rf field for SPINAL64[2] decoupling pulses / kHz	70	70	70	70
^{13}C rf field for 90° and 180° pulses / kHz	-	-	-	70
Rotor syncronized delays for both echos (τ_1 and τ_2) / ms	-	-	-	2.1(τ_1) 2.1(τ_2)
Δt_1 / us	-	-	-	14.3
Number of t_1 increments	-	-	-	962

[a] All recorded on a 9.4 T Bruker Avance III HD solid state NMR spectrometer, using a 4 mm HXY probe in double resonance mode. The corresponding resonance frequencies of ^{1}H and ^{13}C are 400.1 MHz and 100.6 MHz, respectively. [b] "rf" stands for radio-frequency. [c] ^{1}H contact rf field is swept from 70 to 100% of the set ^{1}H rf field linearly with 100 steps during contact pulse.[3]

Table S2. NMR spectra acquisition parameters for activated H-SAPO-34

Experiment	^{13}C CP[a]			^{13}C CP refocused INADEQUATE[a]
Magic angle spinning rate / kHz	10	13.5	14	14
Number of scans	4096	15024	25056	384
Recycle delay / s	3	3	3	3
^{1}H rf field for 90° pulse / kHz	70	83	70	70
CP contact time / ms	2	2	2	2
^{1}H rf amplitude ramp for contact pulse / kHz	ramp70100.100	ramp70100.100	ramp70100.100	ramp70100.100
^{1}H rf field during contact pulse / kHz	60	60	60	60
^{13}C rf field during contact pulse / kHz	56	56	58	46
^{1}H rf field for SPINAL64[2] decoupling pulses / kHz	70	83	70	70
^{13}C rf field for 90° and 180° pulses / kHz	-	-	-	70
Rotor syncronized delays for both echos (τ_1 and τ_2) / ms	-	-	-	2.1(τ_1) 1.4(τ_2)
Δt_1 / us	-	-	-	14.3
Number of t_1 increments	-	-	-	655

[a] All recorded on a 9.4 T Bruker Avance III HD solid state NMR spectrometer, using a 4 mm HXY probe in double resonance mode. The corresponding resonance frequencies of ^{1}H and ^{13}C are 400.1 MHz and 100.6 MHz, respectively.

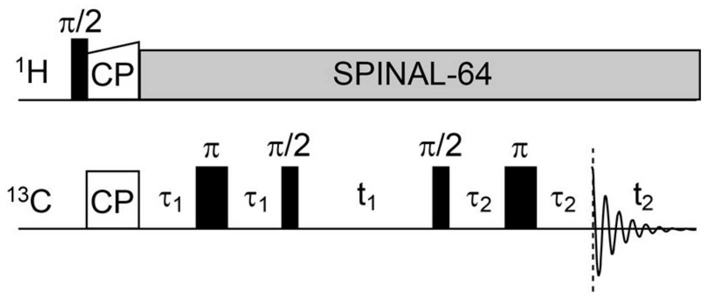

Fig S1. ^{13}C-^{13}C CP refocused INADEQUATE program.[4] τ_1 and τ_2 are synchronized to be an integer number of rotor periods.

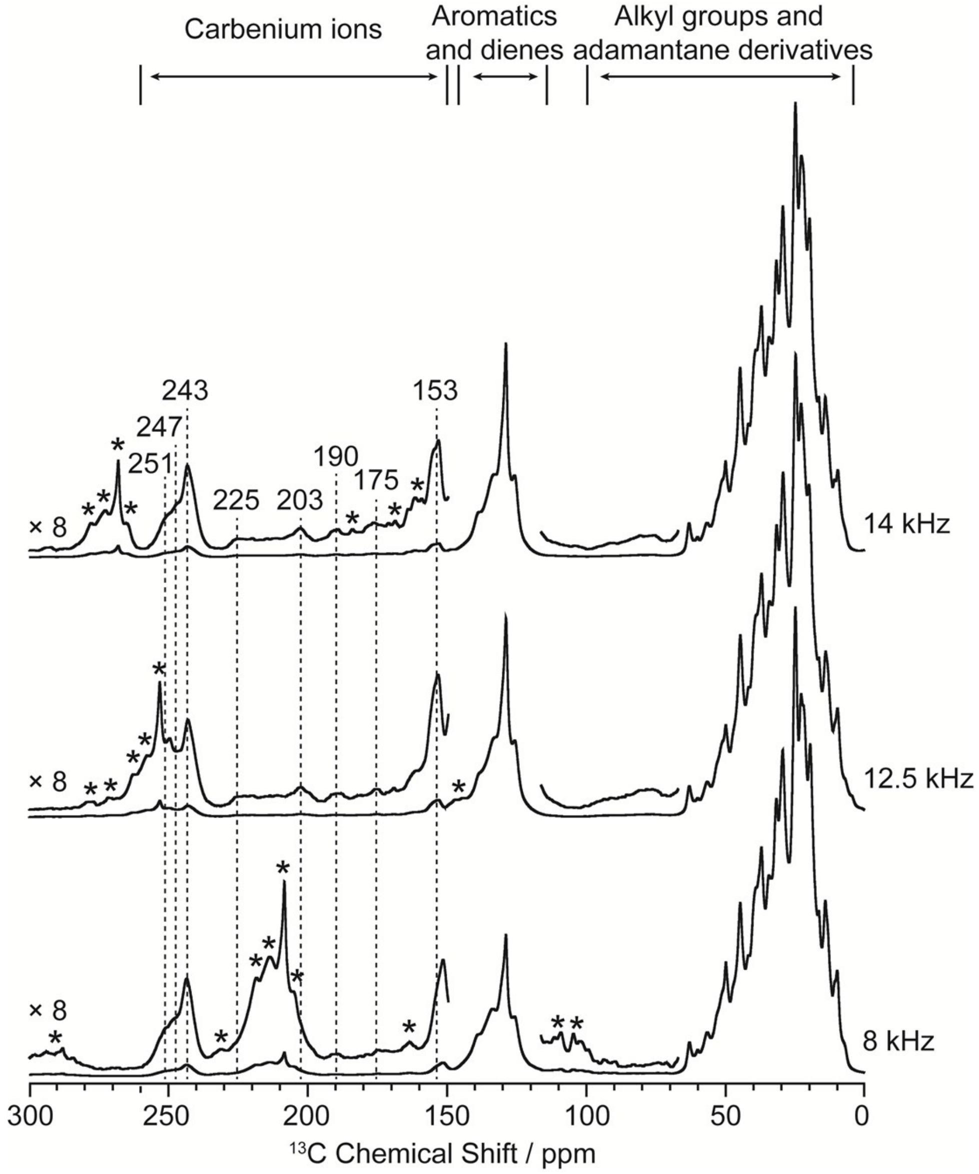

Fig S2. ^{13}C CP spectra of activated H-SSZ-13. Spectra were recorded at 9.4 T and at varied MAS rates of 8 kHz, 12.5 kHz and 14 kHz, respectively. Asterisks (*) denote spinning

sidebands.

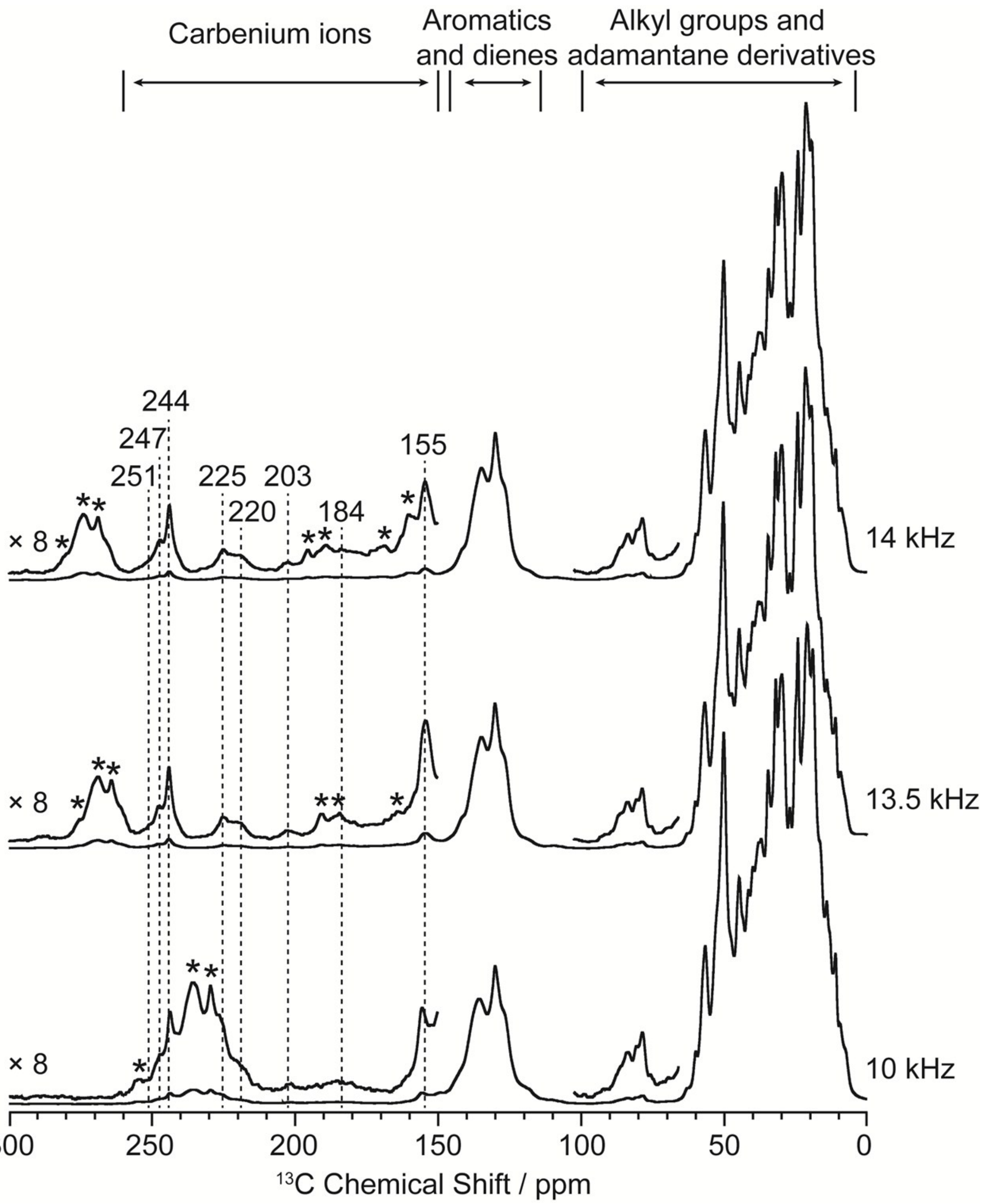

Fig S3. ^{13}C CP spectra of activated H-SAPO-34. Spectra were recorded at 9.4 T and at varied MAS rates of 10 kHz, 13.5 kHz and 14 kHz, respectively. Asterisks (*) denote spinning sidebands.

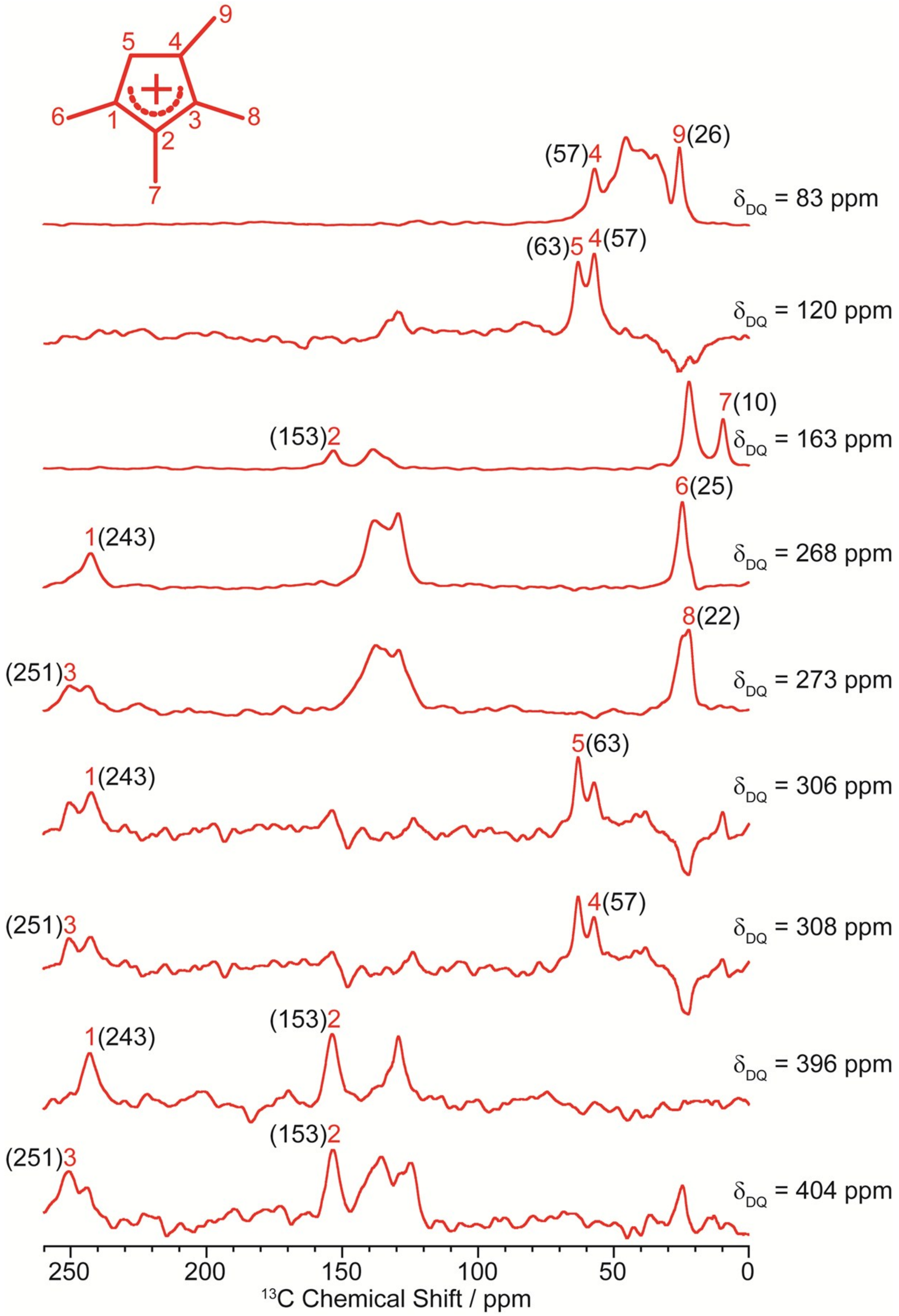

Fig S4. Horizontal traces for 1,2,3,4-tetramethylcyclopentenyl cation **I** in activated H-SSZ-13. The corresponding double quantum frequency δ_{DQ} of each slice is given in the figure. The chemical shifts of different ^{13}C sites are given in the parenthesis. Unlabelled peaks are from other carbenium ions or aromatic species.

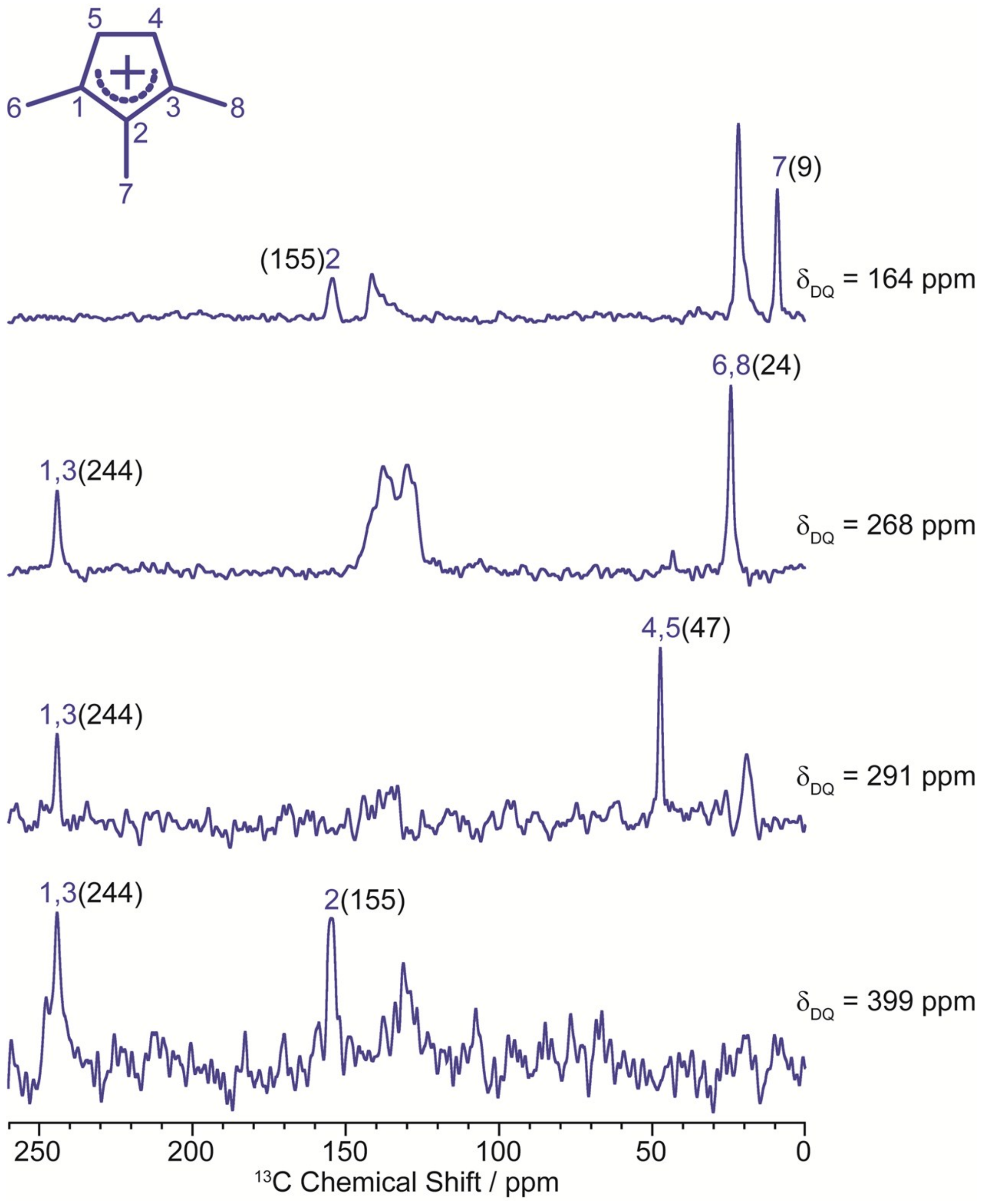

Fig S5. Horizontal traces for 1,2,3-trimethylcyclopentenyl cation **II** in activated H-SAPO-34. The corresponding double quantum frequency δ_{DQ} of each slice is given in the figure. The chemical shifts of different ^{13}C sites are given in the parenthesis. Unlabelled peaks are from other carbenium ions or aromatic species.

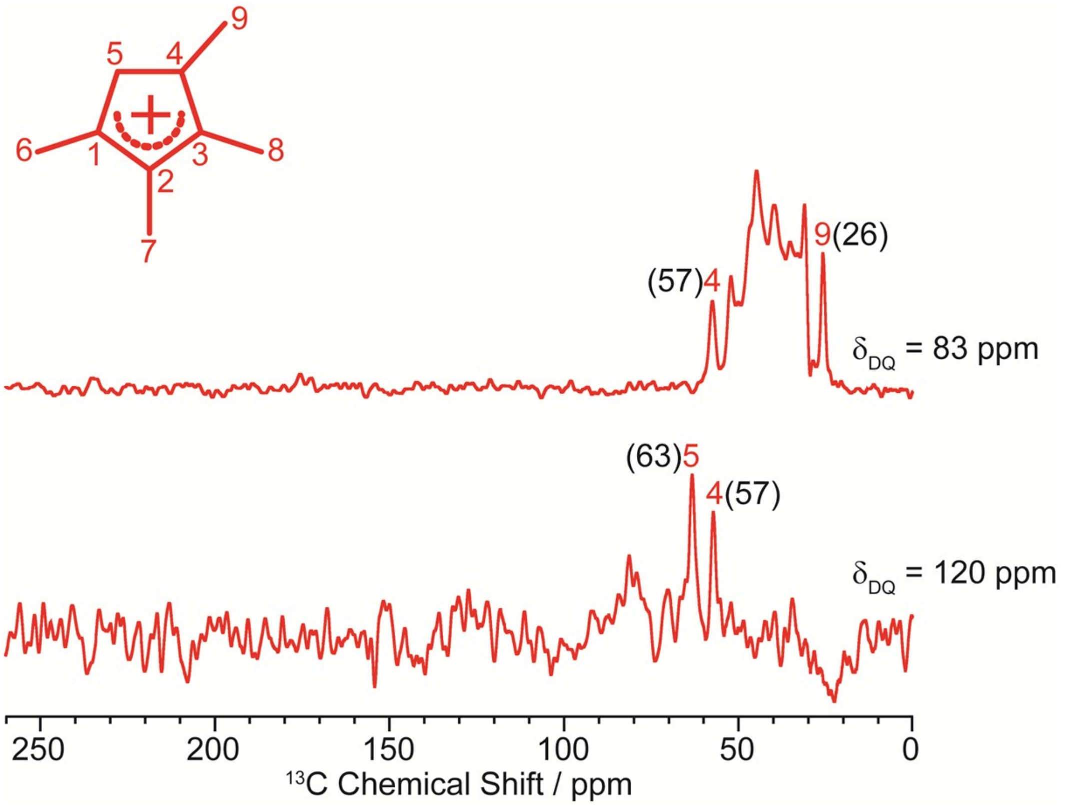

Fig S6. Horizontal traces for 1,2,3,4-tetramethylcyclopentenyl cation **I** in activated H-SAPO-34. The corresponding double quantum frequency δ_{DQ} of each slice is given in the figure. The chemical shifts of different ^{13}C sites are given in the parenthesis. Unlabelled peaks are from other carbenium ions or aromatic species.

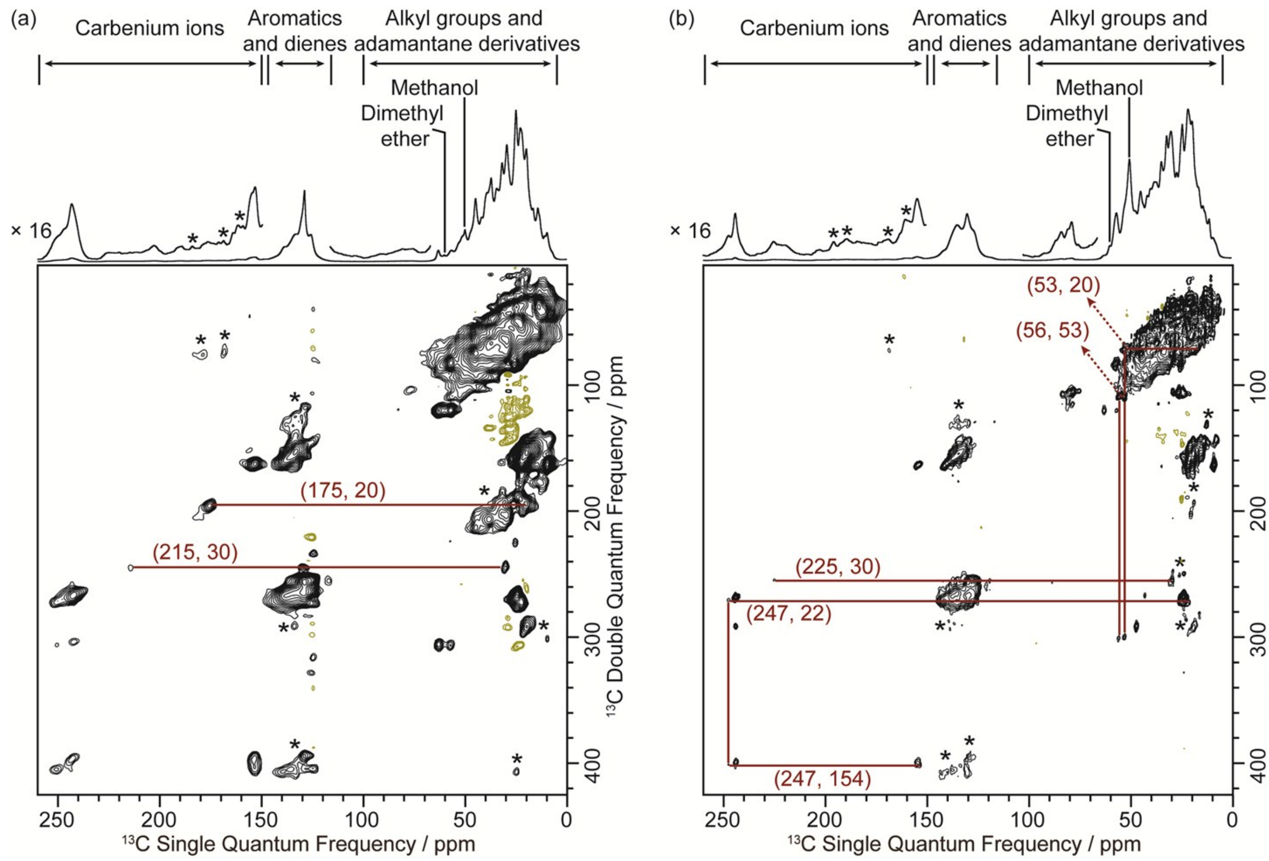

Fig S7. 2D ^{13}C–^{13}C refocused INADEQUATE spectra of activated (a) H-SSZ-13 and (b) H-SAPO-34. Spectra were recorded at 9.4 T and at a MAS rate of 14 kHz. Signals with positive and negative intensities are coded in black and olive, respectively. Asterisks (*) denote spinning sidebands. Partial correlations for carbenium ions including polymethylcyclohexenyl cations,[5–8] cyclopentenyl cations,[9,10] methylnaphthalenium cations[8,11,12] are coded in maroon, and the chemical shifts of the correlated ^{13}C sites are given in the parenthesis.

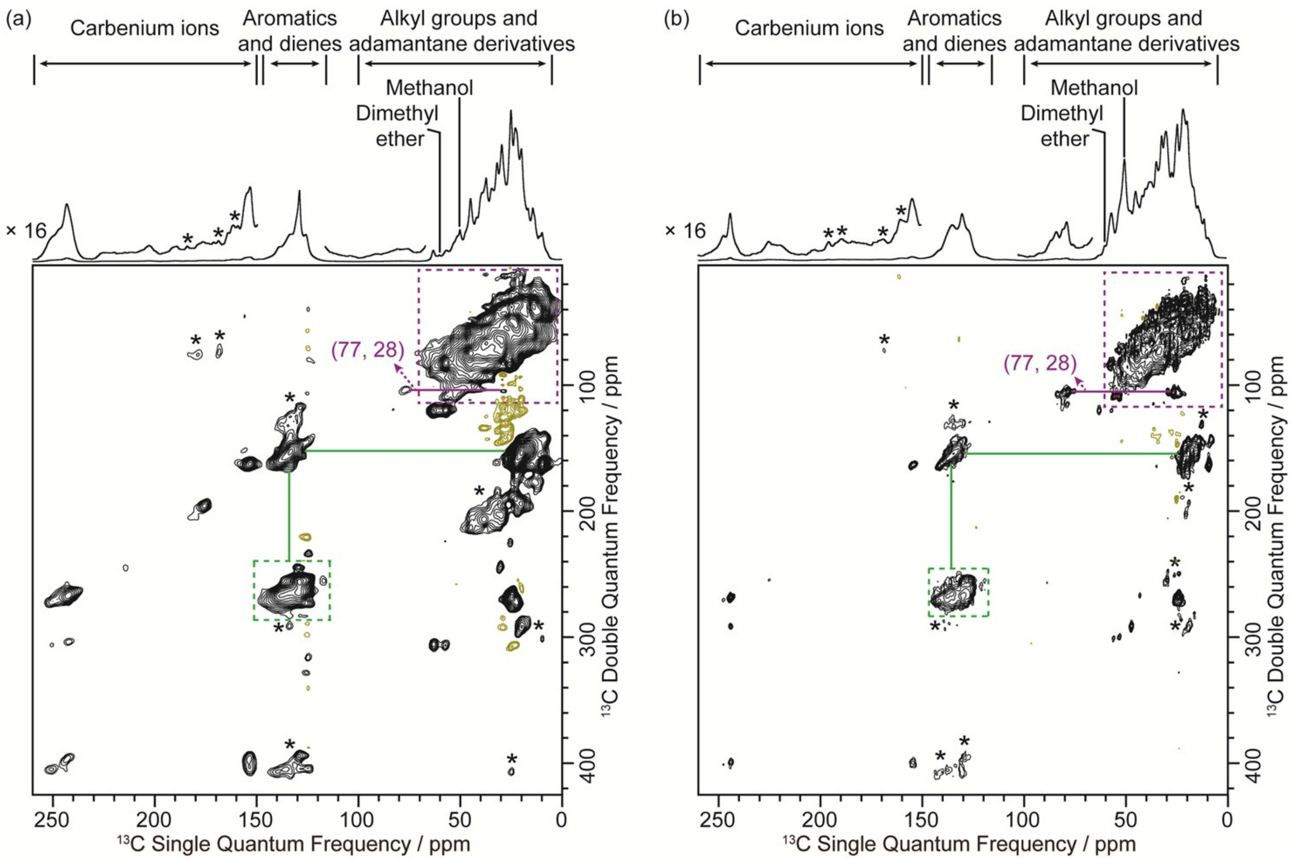

Fig S8. 2D ^{13}C–^{13}C refocused INADEQUATE spectra of activated (a) H-SSZ-13 and (b) H-SAPO-34. Spectra were recorded at 9.4 T and at a MAS rate of 14 kHz. Signals with positive and negative intensities are coded in black and olive, respectively. Asterisks (*) denote spinning sidebands. The correlations in the green dashed box and connected by the green lines belong to the neutral aromatics and dienes. Correlations in the purple dashed box and connected by purple lines are from the alkyl groups in aromatics and carbenium ions and adamantane derivatives.[7–10,13,14] Numbers in the parenthesis are chemical shifts of the correlated ^{13}C sites.

References

1 S. Xu, A. Zheng, Y. Wei, J. Chen, J. Li, Y. Chu, M. Zhang, Q. Wang, Y. Zhou, J. Wang, F. Deng and Z. Liu, *Angew. Chemie Int. Ed.*, 2013, **52**, 11564–11568.

2 R. Fu, S. A. Smith and G. Bodenhausen, *Chem. Phys. Lett.*, 1997, **272**, 361–369.

3 G. Metz, X. Wu and S. O. Smith, *J. Magn. Reson. Ser. A*, 1994, **110**, 219–227.

4 A. Lesage, M. Bardet and L. Emsley, *J. Am. Chem. Soc.*, 1999, **121**, 10987–10993.

5 G. A. Olah and G. Liang, *J. Am. Chem. Soc.*, 1972, **94**, 6434–6441.

6 W. Dai, C. Wang, M. Dyballa, G. Wu, N. Guan, L. Li, Z. Xie and M. Hunger, *ACS Catal.*, 2015, **5**, 317–326.

7 D. Xiao, S. Xu, X. Han, X. Bao, Z. Liu and F. Blanc, *Chem. Sci.*, 2017, **8**, 8309–8314.

8 D. Xiao, S. Xu, N. J. Brownbill, S. Paul, L.-H. Chen, S. Pawsey, F. Aussenac, B.-L. Su, X. Han, X. Bao, Z. Liu and F. Blanc, *Chem. Sci.*, 2018, **9**, 8184–8193.

9 C. Wang, Y. Chu, A. Zheng, J. Xu, Q. Wang, P. Gao, G. Qi, Y. Gong and F. Deng, *Chem. - A Eur. J.*, 2014, **20**, 12432–12443.

10 C. Wang, X. Yi, J. Xu, G. Qi, P. Gao, W. Wang, Y. Chu, Q. Wang, N. Feng, X. Liu, A. Zheng and F. Deng, *Chem. - A Eur. J.*, 2015, **21**, 12061–12068.

11 K. Lammertsma and H. Cerfontain, *J. Am. Chem. Soc.*, 1979, **101**, 3618–3624.

12 K. Lammertsma, *J. Am. Chem. Soc.*, 1981, **103**, 2062–2069.

13 Y. Wei, J. Li, C. Yuan, S. Xu, Y. Zhou, J. Chen, Q. Wang, Q. Zhang and Z. Liu, *Chem. Commun.*, 2012, **48**, 3082–3084.

14 P. R. Seidl, K. Z. Leal and J. D. Yoneda, *J. Phys. Org. Chem.*, 2002, **15**, 801–807.
